# Occult Insulinoma: A 19-Year Diagnostic Delay in a Patient With Recurrent Hypoglycemia

**DOI:** 10.7759/cureus.112066

**Published:** 2026-07-04

**Authors:** Poleth G Medrano-Madrid, Edgar A Espinoza-Beltrán, Jose M Espinoza-Beltrán, Erika M Espinoza-Beltrán, David A Gaspar-Duran

**Affiliations:** 1 Internal Medicine, Servicios de Salud del Instituto Mexicano del Seguro Social para el Bienestar, Hospital General Culiacán, Culiacán, MEX; 2 Radiology, Centro de Investigación y Docencia en Ciencias de la Salud, Hospital Civil de Culiacán, Culiacán, MEX; 3 Endocrinology, Faculty of Medicine, Universidad Autónoma de Sinaloa, Culiacán, MEX; 4 Pathology, Servicios de Salud del Instituto Mexicano del Seguro Social para el Bienestar, Hospital General Culiacán, Culiacán, MEX

**Keywords:** diagnostic delay, endogenous hyperinsulinemic hypoglycemia, insulinoma, pancreatic neuroendocrine tumor, recurrent hypoglycemia, whipple's triad

## Abstract

Recurrent hypoglycemia may present a diagnostic challenge when initial biochemical and imaging studies are inconclusive. We report the case of a 47-year-old man who presented with a 19-year history of recurrent hypoglycemic episodes characterized by diaphoresis, tremor, palpitations, intense hunger, dizziness, blurred vision, and impaired concentration. Initial evaluation documented hypoglycemia; however, insulin and C-peptide concentrations did not meet the diagnostic criteria for endogenous hyperinsulinism, and abdominal computed tomography (CT) failed to identify a pancreatic lesion. Persistent symptoms prompted reevaluation 19 years later, revealing endogenous hyperinsulinemic hypoglycemia and a 16-mm pancreatic body lesion on contrast-enhanced magnetic resonance imaging (MRI). The patient underwent distal pancreatectomy, and histopathological examination confirmed a well-differentiated pancreatic neuroendocrine tumor consistent with insulinoma. Following surgery, hypoglycemic symptoms completely resolved. This case highlights how insulinoma may remain clinically occult for prolonged periods when initial biochemical and imaging findings are nondiagnostic. Persistent or recurrent hypoglycemic symptoms should prompt continued clinical suspicion and periodic reassessment, even after inconclusive investigations, as repeat biochemical evaluation and evolving localization strategies may facilitate definitive diagnosis and treatment.

## Introduction

Insulinoma is the most common functional pancreatic neuroendocrine tumor and a rare cause of endogenous hyperinsulinemic hypoglycemia. Although most lesions are benign and small at presentation, recurrent hypoglycemia may result in substantial morbidity because of autonomic and neuroglycopenic manifestations. The diagnosis is traditionally established by demonstrating Whipple's triad, biochemical evidence of endogenous hyperinsulinism, and localization of the pancreatic lesion using cross-sectional or functional imaging modalities. Despite advances in diagnostic techniques, insulinomas continue to pose a diagnostic challenge because of their low incidence, variable clinical presentation, and frequently small size [1,2].

Patients commonly present with nonspecific symptoms, including diaphoresis, tremor, palpitations, weakness, confusion, visual disturbances, behavioral changes, and loss of consciousness. Because these manifestations overlap with those of neurologic, psychiatric, and metabolic disorders, diagnosis may be delayed for months or even years. Furthermore, small insulinomas may remain undetected on initial imaging studies, particularly during the early stages of the disease, further contributing to diagnostic uncertainty [3,4]. Delayed diagnosis may expose patients to recurrent episodes of severe hypoglycemia, resulting in significant impairment in quality of life and potentially serious clinical consequences [5].

We report the case of an occult pancreatic insulinoma presenting with recurrent hypoglycemia that remained undiagnosed for 19 years before definitive biochemical confirmation, tumor localization, and surgical treatment.

## Case presentation

A 47-year-old man with no significant past medical history presented with a 19-year history of recurrent hypoglycemic episodes characterized by diaphoresis, tremor, palpitations, intense hunger, generalized weakness, dizziness, blurred vision, and impaired concentration. Episodes occurred predominantly in the postprandial period and resolved within minutes after carbohydrate ingestion. During the course of the disease, the lowest documented plasma glucose level was 22 mg/dL. Symptoms consistently coincided with hypoglycemia and improved following glucose administration, fulfilling Whipple's triad.

Initially, episodes occurred approximately once or twice weekly but progressively increased in frequency over time, resulting in significant impairment of daily activities and prompting multiple medical evaluations.

The initial endocrine evaluation revealed hypoglycemia, including a fasting plasma glucose level of 60 mg/dL, accompanied by insulin and C-peptide concentrations that did not meet the biochemical criteria for endogenous hyperinsulinism (Table 1). 

**Table 1:** Biochemical findings at initial evaluation and at the time of definitive diagnosis.

Parameter (Unit)	Initial Evaluation	Follow-up	Reference Range
Plasma glucose (mg/dL)	60	55	70-100
Serum insulin (μU/mL)	2.8	16.6	<3
C-peptide (ng/mL)	1.8	3.5	0.8-3.1
Proinsulin (pmol/L)	-	45	<5
Cortisol (μg/dL)	10.1	17.1	5-25
Hemoglobin A1c (%)	5.0	3.4	4.0-5.6

**Figure 1:**
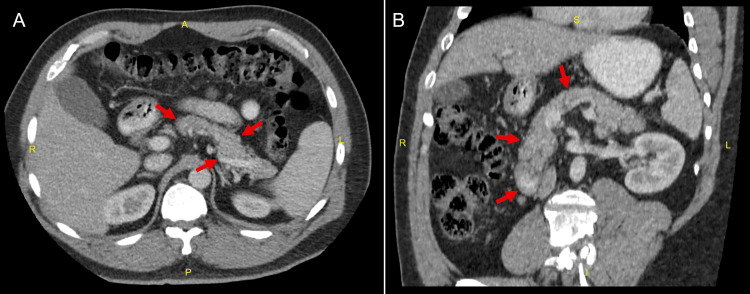
Abdominal CT findings. (A) Axial and (B) coronal images demonstrate a pancreas of normal size and morphology without an identifiable focal pancreatic lesion (red arrows). CT, computed tomography. Orientation markers: A, anterior; P, posterior; R, right; L, left; S, superior; I, inferior.

Contrast-enhanced abdominal computed tomography (CT) demonstrated a pancreas of normal size and morphology without focal lesions, abnormal enhancement, pancreatic duct dilatation, or radiographic evidence of a neuroendocrine neoplasm (Figure 1).

In the absence of definitive biochemical or radiological evidence of insulinoma, the patient was presumed to have reactive hypoglycemia, and treatment with prednisone 5 mg orally once daily was initiated. Partial symptomatic improvement was achieved; however, several attempts to discontinue treatment resulted in recurrence of hypoglycemic episodes. Consequently, prednisone therapy was continued. Despite treatment, intermittent symptoms persisted over the following years. To reduce the frequency of symptomatic episodes, the patient progressively adopted a dietary routine consisting of up to six meals per day, including a bedtime snack. During this period, he gained approximately 10 kg, which was attributed to frequent carbohydrate consumption to prevent or alleviate hypoglycemic episodes.

**Figure 2:**
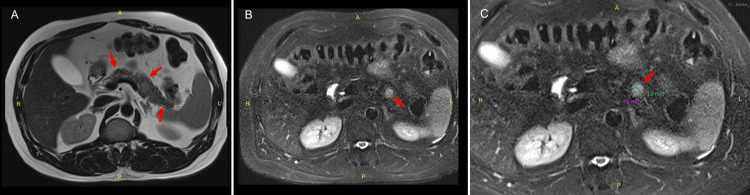
MRI demonstrating a pancreatic body lesion consistent with insulinoma. (A) Axial T2-weighted image without a clearly identifiable focal lesion (red arrows). (B) Axial STIR image demonstrating a focal lesion in the pancreatic body (red arrow). (C) Magnified STIR image showing a well-defined pancreatic body lesion measuring 16 × 13 mm (red arrow). MRI, magnetic resonance imaging; STIR, short tau inversion recovery. Orientation markers: A, anterior; P, posterior; R, right; L, left.

Because of persistent symptoms, the patient underwent reevaluation 19 years after the initial presentation. Laboratory studies demonstrated endogenous hyperinsulinemic hypoglycemia, with elevated insulin, C-peptide, and proinsulin levels documented during a hypoglycemic episode (Table 1). Contrast-enhanced magnetic resonance imaging (MRI) identified a 16-mm solid lesion in the pancreatic body that was highly suggestive of insulinoma (Figure 2). 

**Figure 3:**
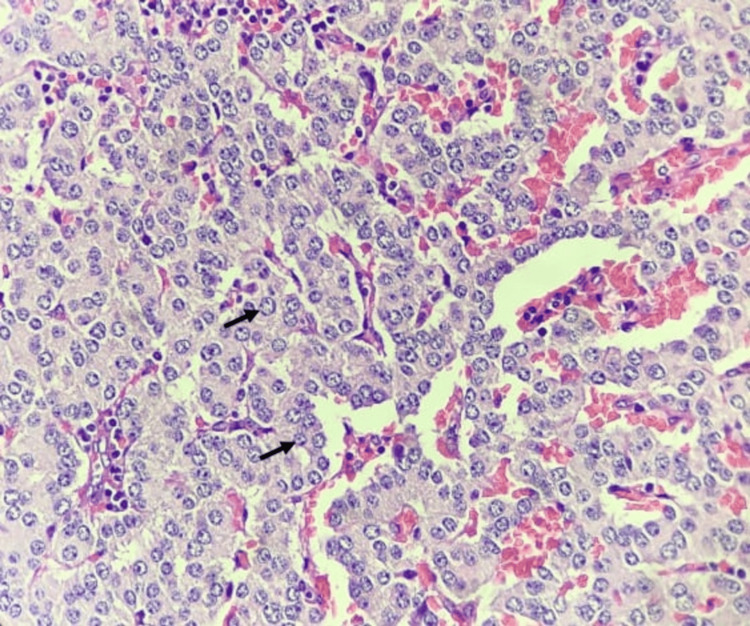
Histopathological findings of the pancreatic lesion. High-power hematoxylin and eosin stain showing a well-differentiated pancreatic neuroendocrine tumor with organoid nests and characteristic salt-and-pepper chromatin (black arrows).

The patient was referred to the surgical oncology service and underwent open distal pancreatectomy. Histopathological examination revealed a well-differentiated pancreatic neuroendocrine tumor, World Health Organization grade 1, measuring 2 cm in greatest dimension, without necrosis, lymphovascular invasion, or perineural invasion, findings consistent with insulinoma (Figure 3). Immunohistochemical studies could not be performed because this technique was unavailable at our institution. Nevertheless, the diagnosis was supported by the concordance of clinical, biochemical, radiological, surgical, and histopathological findings.

At the six-month follow-up, the patient remained free of hypoglycemic episodes, with complete resolution of symptoms and normalization of plasma glucose levels. However, he developed post-pancreatectomy diabetes mellitus and was started on insulin glargine 10 units once daily. During follow-up, he achieved adequate glycemic control without documented recurrent hypoglycemia, recurrence of hypoglycemic symptoms, or other postoperative metabolic complications.

## Discussion

Insulinoma is the most common functional pancreatic neuroendocrine tumor and typically presents with symptoms resulting from recurrent hypoglycemia caused by inappropriate insulin secretion. Patients frequently experience autonomic manifestations such as diaphoresis, tremor, palpitations, and hunger, as well as neuroglycopenic symptoms including confusion, visual disturbances, behavioral changes, and loss of consciousness [3]. In the present case, the patient experienced many of these characteristic manifestations and developed documented severe hypoglycemia that improved following carbohydrate ingestion. These findings fulfilled Whipple's triad, which remains the cornerstone for the evaluation of hypoglycemic disorders and the recognition of endogenous hyperinsulinism [6]. Furthermore, the patient's progressive weight gain likely reflected chronic compensatory carbohydrate intake aimed at preventing or alleviating hypoglycemic episodes.

Despite a clinical presentation highly suggestive of insulinoma, the diagnosis was not established during the initial evaluation. Although hypoglycemia was documented during the initial evaluation, insulin and C-peptide concentrations did not meet the diagnostic criteria for endogenous hyperinsulinism, and abdominal computed tomography failed to identify a pancreatic lesion. Importantly, insulin secretion appeared appropriately suppressed relative to the degree of hypoglycemia during the initial evaluation, in contrast to the unequivocal hyperinsulinemia documented at the time of definitive diagnosis. Such findings are not entirely unexpected, as insulinomas are frequently small tumors that may evade detection on conventional imaging studies, particularly during the early stages of the disease [1,2].

Furthermore, biochemical confirmation may be challenging when laboratory evaluation is not obtained during a representative spontaneous hypoglycemic episode. In addition, insulinomas may exhibit intermittent insulin secretion, resulting in biochemical findings that do not initially fulfill diagnostic criteria despite clinically significant hypoglycemia. The subsequent demonstration of elevated insulin, C-peptide, and proinsulin levels during documented hypoglycemia in our patient illustrates the dynamic nature of endogenous hyperinsulinism and highlights the importance of repeat biochemical evaluation when clinical suspicion persists [1,4]. At that stage, the available biochemical and imaging findings were insufficient to establish a diagnosis of insulinoma despite the presence of recurrent, predominantly postprandial hypoglycemic symptoms. An additional strength of this case is the availability of biochemical data obtained both at the initial evaluation and at the time of definitive diagnosis, illustrating the evolution from nondiagnostic findings to unequivocal evidence of endogenous hyperinsulinism. This case also highlights the limitations of conventional cross-sectional imaging, as abdominal computed tomography failed to identify the lesion, whereas contrast-enhanced magnetic resonance imaging subsequently localized a 16-mm pancreatic lesion.

When conventional cross-sectional imaging is inconclusive despite persistent clinical suspicion of insulinoma, additional localization techniques should be considered. Endoscopic ultrasound (EUS) may identify small pancreatic insulinomas not visualized by CT or MRI and has demonstrated particular value in patients with persistent clinical and biochemical suspicion despite negative conventional imaging [7]. Selective arterial calcium stimulation with hepatic venous sampling (SACST) remains a valuable invasive localization technique, particularly in patients with biochemically confirmed endogenous hyperinsulinemic hypoglycemia and negative or equivocal imaging studies [8]. Functional molecular imaging, including gallium-68 DOTA-(Tyr3)-octreotate (^68Ga-DOTATATE) positron emission tomography/computed tomography (PET/CT), can also serve as a complementary localization modality, although its sensitivity may be limited in some benign insulinomas because of variable somatostatin receptor subtype 2 expression [9]. Therefore, these techniques may facilitate tumor localization and surgical planning when conventional imaging fails to identify the lesion.

The prolonged interval between symptom onset and definitive diagnosis represents the most notable aspect of this case. Although most insulinomas are diagnosed within a considerably shorter time frame, delayed diagnosis remains a recognized clinical challenge, particularly in patients with nonspecific symptoms, initially inconclusive biochemical findings, or negative imaging studies. Previous reports have described diagnostic delays ranging from five years to several decades, often related to nonspecific clinical manifestations, initially inconclusive investigations, or delayed tumor localization [10-12]. Similar to these reports, our patient remained symptomatic for nearly two decades before biochemical confirmation of endogenous hyperinsulinism and localization of the pancreatic lesion. Following the initial evaluation and initiation of prednisone therapy, the patient gradually adapted to recurrent hypoglycemia by consuming up to six meals per day, including a bedtime snack, to reduce the frequency and severity of symptomatic episodes. Although he remained able to continue working and perform his usual daily activities, this adaptive strategy became part of his daily routine despite persistent concern about recurrent hypoglycemia. Compared with previously published cases, this report further emphasizes the importance of maintaining a high index of suspicion when evaluating patients with recurrent unexplained hypoglycemia.

An important lesson from this case is that negative imaging studies and initially inconclusive biochemical findings should not definitively exclude insulinoma when the clinical history remains strongly suggestive of recurrent hypoglycemia. The persistence of symptoms despite medical therapy, recurrent episodes over many years, and progressive clinical impact ultimately justified reassessment, which led to the diagnosis. This observation is consistent with previous reports emphasizing the importance of maintaining clinical suspicion and repeating diagnostic investigations when symptoms remain unexplained [4,5]. Periodic reassessment should therefore be considered in patients with recurrent unexplained hypoglycemia, particularly when clinical suspicion remains high despite initially negative investigations.

A limitation of this report is the absence of immunohistochemical confirmation because these studies were unavailable at our institution. Nevertheless, the diagnosis was supported by the concordance of the patient's clinical presentation, biochemical evidence of endogenous hyperinsulinemic hypoglycemia, radiologic localization, surgical findings, and the characteristic histopathologic features of a well-differentiated pancreatic neuroendocrine tumor. The present case highlights how insulinoma may remain clinically occult for prolonged periods despite repeated medical evaluations. Recognition of persistent symptom patterns, coupled with reassessment using updated biochemical and imaging modalities, may ultimately allow definitive diagnosis and treatment, resulting in complete resolution of hypoglycemic symptoms.

## Conclusions

This case highlights the diagnostic challenges associated with insulinoma, particularly when initial biochemical studies and conventional imaging findings are nondiagnostic. Despite a clinical presentation characterized by recurrent, predominantly postprandial hypoglycemia, definitive diagnosis was delayed for 19 years until repeat biochemical evaluation and imaging identified a pancreatic lesion. Persistent or recurrent hypoglycemic symptoms should prompt continued clinical suspicion and periodic reassessment, even when initial investigations are inconclusive. Appropriate repeat biochemical evaluation and the use of complementary localization techniques, when clinically indicated, may facilitate earlier diagnosis and definitive treatment, preventing years of avoidable morbidity.
